# Health service utilization by indigenous cancer patients in Queensland: a descriptive study

**DOI:** 10.1186/1475-9276-11-57

**Published:** 2012-10-10

**Authors:** Christina M Bernardes, Lisa J Whop, Gail Garvey, Patricia C Valery

**Affiliations:** 1 Epidemiology and Health Systems Division, Menzies School of Health Research, Charles Darwin University, Adelaide Street, PO Box 10639, Brisbane, QLD, 4000, Australia

**Keywords:** Indigenous Australian, Cancer, Health services utilization

## Abstract

**Introduction:**

Indigenous Australians experience more aggressive cancers and higher cancer mortality rates than other Australians. Cancer patients undergoing treatment are likely to access health services (e.g. social worker, cancer helpline, pain management services). To date Indigenous cancer patients’ use of these services is limited. This paper describes the use of health services by Indigenous cancer patients.

**Methods:**

Indigenous cancer patients receiving treatment were recruited at four major Queensland public hospitals (Royal Brisbane Women’s Hospital, Princess Alexandra, Cairns Base Hospital and Townsville Hospital). Participants were invited to complete a structured questionnaire during a face-to-face interview which sought information about their use of community and allied health services.

**Results:**

Of the 157 patients interviewed most were women (54.1%), of Aboriginal descent (73.9%), lived outer regional areas (40.1%) and had a mean age of 52.2 years. The most frequent cancer types were breast cancer (22.3%), blood related (14.0%), lung (12.1%) and gastroenterological (10.8%). More than half of the participants reported using at least one of the ‘Indigenous Health Worker/Services’ (76.4%), ‘Allied Health Workers/Services’ (72.6%) and ‘Information Sources’ (70.7%). Younger participants 19–39 years were more likely to use information sources (81.0%) than older participants who more commonly used community services (48.8%). The cancer patients used a median of three health services groups while receiving cancer treatment.

**Conclusions:**

Indigenous cancer patients used a range of health services whilst receiving treatment. Indigenous Health Workers/Services and Allied Health Workers/Services were the most commonly used services. However, there is a need for further systematic investigation into the health service utilization by Indigenous cancer patients.

## Introduction

Cancer has been recently identified as a leading cause of death for Aboriginal and Torres Strait Islander people (hereafter referred to as Indigenous Australians) [1]. In Queensland, mortality rates are up to 36% higher for Indigenous people in comparison to non-Indigenous people with cancer [2]. Cancer survival is also poorer [3]. In a recent study comparing survival rates of Indigenous and non-Indigenous people with cancer, Indigenous people had 50% higher mortality in the first 12 months after diagnosis [4]. The reasons for the poorer outcomes are complex and not yet fully understood. Less access to early cancer detection programs [3,5], less treatment [6] and higher rates of comorbidities [3] have been cited as possible reasons that may explain the differences in cancer outcomes.

Despite advances in cancer care, recommended medical treatments and screening procedures are not provided to all patients who are likely to benefit [7]. Disparities between best-evidence practice and existing care means cancer patients continue to receive different levels of care, resulting in differences in important outcomes [7,8]. Research has shown that differences in healthcare access and quality are important mediators of survival disparities between many populations of patients with cancer [4]. Health system factors may impact both at the level of treatment decisions and processes, and at more structural level such as the location, resourcing and accessibility of health care facilities [9]. Investigation on satisfaction of cancer patients with their care has shown that although a majority of cancer patients report being satisfied with clinical aspects of their care, they are less satisfied with the information and support they receive [10]. In particular, dissatisfaction has been expressed in relation to the information received about the disease, treatment, side effects and their control, and the support patients and their family receive after returning home [11].

Regarding the access to services, Indigenous people are over ten-times more likely than non-Indigenous Australian to live in remote areas, which makes access to cancer treatment services more challenging [5,12]. The distribution of this population however, varies from one state to other, for example, while in Queensland, it was estimated that 22% of the Indigenous population lived in remote areas in New South Wales only 5% [13]. Indigenous people are equally as likely as non-Indigenous people to have a consultation with a general practitioner, more than twice as likely to visit the casualty or outpatients department of a hospital, and half as likely have a consultation with a dentist [14]. To date there is limited evidence regarding health service utilization by Indigenous cancer patients and the factors that may affect the use of these services. For Indigenous cancer patients, additional logistic and cultural factors appear to affect their access to cancer health services. Logistical factors may include but are not limited to difficulties in communication between medical staff and patients, the availability of transport, the distance to travel required for diagnosis, treatment and follow-up care, accommodation, financial burden and conflicting family priorities. Some cultural factors that may impact on Indigenous patients access to services include a shortage of Indigenous staff at the service and fear and distrust associated with accessing a more ‘mainstream’ service where staff maybe less prepared to deal with cultural differences [1,15]. Indigenous people’s perception about cancer may also affect their utilisation of services [5]. Research exploring Indigenous people’s views of cancer reported that their fear of cancer and perception of cancer as a death sentence contributes to the belief that treatment is beyond the control of medicine [16,17]. Whilst it is known that health services are important in assisting cancer patients through their cancer journey (e.g. social worker, cancer helpline, pain management services), little is known of how Indigenous cancer patients’ use such services. Here we describe the use of community and allied health services by Indigenous cancer patients undergoing treatment in Queensland.

## Methods

### Study setting and participants

This study is a component of a much larger study in Queensland that is investigating the supportive care needs of Indigenous adult cancer patients undergoing cancer treatment (Supportive Care Needs Study). Participants were recruited from four major treating hospitals (2010-present). Here we included participants recruited during the first 14 months of this study (September 2010 to November 2011).

Indigenous adult patients diagnosed with cancer (any type), hospitalized or attending a hospital outpatient clinic for cancer treatment or follow-up care were recruited from the Royal Brisbane Women’s Hospital, Princess Alexandra, Cairns Base and Townsville hospitals. Patients were eligible to be included in the study if they were receiving cancer treatment (chemotherapy, radiotherapy, or surgery) or had recently completed cancer treatment (no longer than 30 days before enrolling in the study), were mentally capable and physically well enough to be interviewed.

Hospital staff (Indigenous Liaison Officers or nursing staff) made initial contact with potential participants (a study flyer was handed to patients). If the patient agreed to be contacted, the project research assistant (RA) then contacted participants to provide more information about the study, answer any question participants had about the study, confirm Indigenous status, and obtain written consent to participate. Participants were also ascertained using hospital monthly reports of discharged Indigenous cancer patients and daily lists of inpatients. All patients identified on these lists were then cross-checked with the hospitals’ Indigenous Liaison Officer’s lists of Indigenous patients. Patients not already approached about the study from these lists were then contacted by the Indigenous Liaison Officer to seek their interest in participation.

Data was collected using a structured questionnaire and delivered via face-to-face interviews conducted in English at a place convenient to both participants and the interviewer. Six interviewers were of Aboriginal and/or Torres Strait Islander descent and 3 are non-Indigenous. Interviewers received standardised training to conduct the interviews and the first few interviews were taped and monitored by the study’s project manager for consistency across four study sites. The structured-questionnaire included the following question: “Have you accessed any of the following community or allied health services for support with your cancer?”. Participants were then asked to identify which of the 26 stated services they accessed when the interviewer read aloud the options (see Additional file 1). Participants could also report any other services accessed that were not listed. Other relevant data included: demographic characteristics (age, marital status, education, employment, place of residence), cancer type, and cancer treatment (surgery, chemotherapy, radiation or others).

The 26 health services were grouped into the following categories: Group 1 – Indigenous Health Workers/Services; Group 2 – Information Sources; Group 3 – Support Services; Group 4 – Community Services; Group 5 – Allied Health Workers/Services and Group 6 – Others (where patients could specify a service not otherwise listed). Patients were able to select more than one service.

Using postcode, patients’ place of residence (referred here as ‘accessibility’ to health services) was determined on the basis of the Australian Standard Geographical Classification (ASGC) and by the Accessibility/Remoteness Index of Australia (ARIA) [18]. Due to the small number of patients in some categories, it was necessary to aggregate the ARIA from five categories (Major city, Inner city, outer regional, remote and very remote) into two categories. ‘Major city’, ‘Inner regional’ and ‘Outer regional’ were grouped and referred to here as ‘accessible’, ‘remote’ and ‘very remote’ were grouped and referred to here as ‘remote’. Socioeconomic status was defined using the Socioeconomic Indexes for Areas (SEIFA), the Index of Relative Socioeconomic Advantage and Disadvantage (IRSAD) [19]. SEIFA was used to classify the patients’ usual area of residence into groups ranging from ‘most advantaged’, ‘advantaged’, ‘intermediate advantage’, ‘low to intermediate advantage’ and ‘most disadvantaged’. These categories were further regrouped into ‘advantaged’ (groups ‘most advantaged’, ‘advantaged’, ‘intermediate advantage’) and ‘disadvantaged’ (groups ‘low to intermediate advantage’ and ‘most disadvantaged’).

The participants ‘accessibility’ to health services was determined on the basis of the Australian Standard Geographical Classification (ASGC) and by the Accessibility/Remoteness Index of Australia (ARIA) [18] and socioeconomic status was defined using the Socioeconomic Indexes for Areas (SEIFA), and the Index of Relative Socioeconomic Advantage and Disadvantage (IRSAD) [19].

Data analysis was conducted using SPSS Inc version 17.11 (College Station TX: StatCorp LP; 2009). Chi-squared tests were used to test proportions (Fisher’s exact test was used when cell counts were less than 5). Statistical significance was set at alpha = 0.05.

Approval for this study was obtained from the Human Ethics Committees at the Queensland Institute of Medical Research, the Australian National University, and the four participating hospitals.

## Results

**Figure 1:**
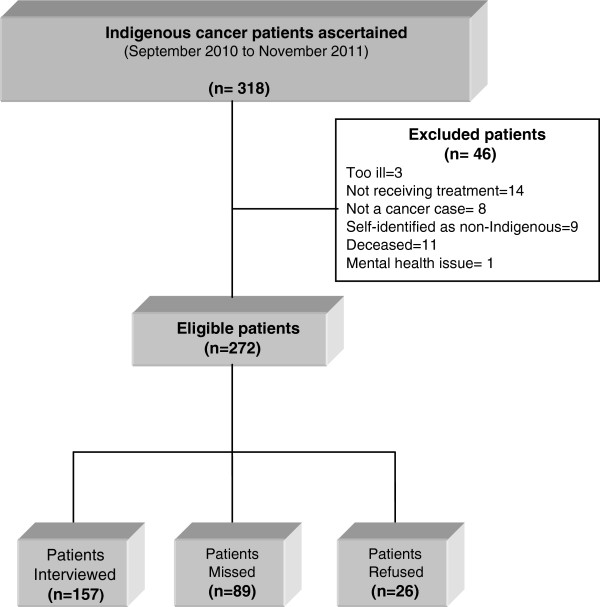
Patient flowchart through the study.

A total of 318 Indigenous cancer patients were ascertained (admitted to these hospitals or attended cancer outpatient clinics) from September 2010 to November 2011: 272 (85.5%) were eligible for the study, 46 (14.5%) were excluded (too sick to be interviewed, currently not receiving any treatment, self-identify as non-Indigenous, mental health issue or deceased) please refer to Figure 1, Patient flowchart through the study. One hundred and eighty three were invited to take part in the study: 157 were interviewed (57.7% response rate) and 26 refused (9.6%). ‘Patients missed’ (n=89, 32.7%).

Of the 157 participants included in the study, over half were women (54.1%), aged between 40 to 59 years old. The mean age of participants was 52 years (SD=13.277, range 19 to 78 years). Regarding Indigenous status, most participants self identified as Aboriginal descent (73.9%), followed by Torres Strait Islander (14.6%), Aboriginal and Torres Strait Islander (5.1%) and the remaining participants reported being Aboriginal and South Sea Islander, Torres Strait Islander and Papa New Guinean and of Aboriginal and European descent (6.4%). Fewer participants reported having higher than primary school level of education (63.2% had primary school, 29.9% had high school or more) however over half of the participants (55.4%) were classified as having advantaged socio-economic status. Forty percent of the participants lived in an outer regional area; 51% (n=80) had to travel away from home to receive their cancer treatment, from rural and remote areas to the treating hospitals in Brisbane, Townsville and Cairns. Most participants reported speaking mainly English at home (84.5%) and no other Aboriginal or Torres Strait Islander language (53.5%). The most frequent cancer groups reported by participants were breast (22.3%), blood related (14.0%), lung cancer (12.0%), gastroenterological (10.8%), head and neck (9.6%), male genital organs (9.6%) and gynaecological (8.9%). At the time of interview 67.5% of patients were receiving treatment as outpatients.

The ‘Patients missed’ (n=89, 32.7%) were: mostly women (68.5%), mean age of 49 years (SD= 14.984, range 23 to 92 years), diagnosed with gynaecological (17.9%), breast cancers (15.7%), gastroenterological (11.2%) and blood related (10.1%). There was missing data for two patients regarding their age and 15 patients regarding their cancer type.

The health services utilisation reported by participants is presented in Table 1. Overall 97% of Indigenous cancer patients reported utilized at least one community or allied health service for support with their cancer. Approximately, three quarters of the patients interviewed indicated they used at least one of the ‘Indigenous Health Worker/Services’ (76.4%), ‘Allied Health Workers/Services’ (72.6%) and ‘Information Sources’ (70.7%). Almost half of the participants reported they had accessed ‘Community Services’ (44.6%), and the ‘Support Service’ group was least reported (29.3%). A small proportion of participants (15.3%) identified services not listed (other services). An Indigenous Health Liaison Officer (68.8%), a dietician (42.0%) and a social worker (38.2%) were the most frequent health workers accessed by participants. Brochures (66.9%) were the most common information source amongst participants. Whilst the Cancer Helpline was the most commonly reported ‘Support Service’, it was utilized by only 12.1% of participants. Among the ‘Community Services’, nearly a third of the participants indicated transport (33.8%) as the main service used.

**Table 1:** Reported health services utilization by Indigenous cancer patients in Queensland

Health services groups*	**Number**	**(%)**
**Group 1 - Indigenous Health Workers/Services**	120	(76.4)
Aboriginal Health Service	55	(35.0)
Indigenous Health Liaison Officer	108	(68.8)
Traditional Indigenous Practitioner	3	(1.9)
**Group 2 - Information Sources**	111	(70.7)
Information sheets/ Brochures	105	(66.9)
Internet information	34	(21.7)
Education Program/workshop	29	(18.5)
**Group 3 - Support Services**	46	(29.3)
Cancer helpline	19	(12.1)
Chaplain	17	(10.8)
Community-based support group	8	(5.1)
Internet-based support group	5	(3.2)
Peer support	7	(4.5)
Tele-based cancer counselling	3	(1.9)
**Group 4 - Community Services**	70	(44.6)
Home and Community Care Services (HACC)	31	(19.7)
Respite Care	4	(2.5)
Transport	53	(33.8)
**Group 5 - Allied Health Workers/Services**	114	(72.6)
Complementary medicine practitioner	5	(3.2)
Community Health Nurse	34	(21.7)
Dietician	66	(42.0)
Exercise physiologist	10	(6.4)
Mental health team	03	(1.9)
Pain specialist	18	(11.5)
Physiotherapist	36	(22.9)
Psychologist	15	(9.6)
Psychiatrist	3	(1.9)
Relaxation/meditation class	6	(3.8)
Social worker	60	(38.2)
**Group 6 - Others**	24	(15.3)
Cancer Care Coordinators/Cancer Council/Cancer Care Queensland	20	(12.7)
General practitioner	3	(1.9)
Occupational Therapist	2	(1.3)
Speech Therapist	2	(1.3)
Red Cross	1	(0.6)
Homeless Organization	1	(0.6)
Elders	1	(0.6)
Breast Care Association	1	(0.6)
**Total**	157	(100.0)

*The patients could indicate the use of more than one health service.

**Percentage calculated over total number of patients (157).

There was no significant difference on the use of services by cancer type (Table 2). However, patients with breast and blood related cancers appeared use a wider range of health services groups (most patients (>74%) used services from at least 3 health services groups). Overall, each participant reported using a median number of three health services with a range of none to six services.

**Table 2:** Number and proportion of Indigenous cancer patients that used health services, by cancer type, in Queensland

**Type of cancer (ICD 10)**	**Health services groups**														
	**Number cancer patients**	**Indigenous health workers/ services**		**Information sources**		**Support services**		**Community services**		**Allied health workers/ services**		**Others**		Overall use of health services**	
	N	N*	(%)	N*	(%)	N*	%	N*	%	N*	%	N*	(%)	Median	Range
Breast (C50)	35	26	(74)	28	(80)	12	(34)	16	(46)	26	(74)	5	(14)	3	1-6
Blood related (lymphoid/haematopoietic and related tissue) (C81-C96)	22	19	(86)	18	(82)	8	(36)	12	(54)	19	(86)	-	-	4	0-5
Lung (C30-C39)	19	16	(84)	13	(68)	6	(32)	10	(53)	13	(68)	4	(21)	3	1-6
Gastroenterological (C15-C26)	17	14	(82)	10	(59)	3	(18)	6	(35)	13	(76)	3	(18)	3	0-6
Lip, oral cavity and pharynx (C00-C14)	15	13	(87)	8	(53)	3	(20)	10	(67)	12	(80)	2	(13)	3	1-5
Male genital organs (C60-C63)	15	9	(60)	8	(53)	5	(33)	8	(53)	11	(73)	2	(13)	3	0-4
Gynaecological (C51-C58)	14	8	(57)	11	(79)	4	(29)	4	(29)	10	(71)	4	(29)	3	0-4
Thyroid/other endocrine glands (C73-C75)	06	2	(33)	4	(67)	2	(33)	-	-	2	(33)	2	(33)	1	1-5
Others	19	15	(79)	15	(79)	5	(26)	4	(21)	10	(53)	3	(16)	-	-
p value***		0.375		0.187		0.876		0.120		0.501		0.277		-	
Total	157	120		111		46		70		114		24			

*Number of patients with a specific cancer type who reported using this service (% of patients with a specific cancer type who reported using this service).

**Patients could indicate the use of more than one health service.

***p value for the difference in use of each health service group by cancer type.

Health service utilization was stratified by gender, age, education, SEIFA and ARIA were analysed (Table 3). In general, there was no significant difference in health service utilization by gender or age group. The only exception was the use of the ‘Information Sources Group’ where females were more likely to report using such services (p= 0.038). Individuals with high school education level or more were more likely to use services grouped under ‘Information Sources’ (p= 0.001) and ‘Support Services (p= 0.030) than those with less than high school education. Furthermore, a higher proportion of patients with lower education level used services grouped under ‘Community Services’ (p= 0.066), although not statistically significant. There was no significant difference on service utilization by socioeconomic status. Patients living in remote areas were just as likely as patients in accessible areas to use health services (p= 0.349).

**Table 3:** Demographics, socioeconomic status (SEIFA), area of remoteness (ARIA) and health service utilization by Indigenous cancer patients in Queensland

**Characteristics**		**Health services groups**																							
	**Total number of patients**	**Indigenous health workers/ services**				**Information sources**				**Support services**				**Community services**				**Allied Health workers/serv.**				**Others**			
		**Use**		**Did not**		**Use**		**Did not**		**Use**		**Did not**		**Use**		**Did not**		**Use**		**Did not**		**Use**		**Did not**	
	**N**	**N**	**(%)**	**N**	**(%)**	**N**	**(%)**	**N**	**(%)**	**N**	**(%)**	**N**	**(%)**	**N**	**(%)**	**N**	**(%)**	**N**	**(%)**	**N**	**(%)**	**N**	**(%)**	**N**	**(%)**
**Gender**																									
Male	72	59	(82)	13	(18)	45	(63)	27	(38)	17	(24)	55	(76)	33	(46)	39	(54)	52	(72)	20	(28)	09	(13)	63	(88)
Female	85	61	(72)	24	(28)	66	(78)	19	(22)	29	(34)	56	(66)	37	(44)	48	(56)	62	(73)	23	(27)	15	(18)	70	(82)
p value		0.134				0.038				0.150				0.772				0.920				0.372			
**Age group**																									
19-39 years	26	20	(77)	06	(23)	21	(81)	05	(19)	08	(31)	18	(69)	10	(38)	16	(62)	22	(85)	04	(15)	06	(23)	20	(77)
40-59 years	86	67	(78)	19	(22)	62	(72)	24	(28)	26	(30)	60	(70)	38	(44)	48	(56)	62	(72)	24	(28)	14	(16)	72	(84)
≥60 years	45	33	(73)	12	(27)	28	(62)	17	(38)	12	(27)	33	(73)	22	(49)	23	(51)	30	(67)	15	(33)	04	(09)	41	(91)
**p value**		0.841				0.233				0.899				0.692				0.260				0.258			
Education level*																									
Less than high school	98	73	(74)	25	(26)	61	(62)	37	(38)	23	(23)	75	(77)	50	(51)	48	(49)	68	(69)	30	(31)	13	(13)	85	(87)
High school and more	57	45	(79)	12	(21)	50	(88)	07	(12)	23	(40)	34	(60)	20	(35)	37	(65)	44	(77)	13	(23)	11	(19)	46	(81)
p value		0.530				0.001				0.027				0.055				0.295				0.317			
**Socioeconomic status (SEIFA)**																									
Disadvantaged	70	52	(74)	18	(26)	49	(70)	21	(30)	18	(26)	52	(74)	36	(51)	34	(49)	51	(73)	19	(27)	08	(11)	62	(89)
Advantaged	87	68	(78)	19	(22)	62	(71)	25	(29)	28	(32)	59	(68)	34	(39)	53	(61)	63	(72)	24	(28)	16	(18)	71	(82)
p value		0.570				0.863				0.376				0.122				0.951				0.228			
**Area of remoteness (ARIA)**																									
Accessible	126	94	(75)	32	(25)	88	(70)	38	(30)	40	(32)	86	(68)	56	(44)	70	(56)	91	(72)	35	(28)	20	(16)	106	(84)
Remote	31	26	(84)	5	(16)	23	(74)	8	(26)	06	(19)	25	(81)	14	(45)	17	(55)	23	(74)	08	(26)	04	(13)	27	(87)
**p value**		0.276				0.633				0.174				0.943				0.825				0.681			

## Discussion

In this study, information about community and allied health service use by Indigenous people diagnosed with cancer was collected. Overall, our findings indicate that in Queensland, Indigenous cancer patients accessed multiple community and allied health services. To our knowledge there is no study amongst non-Indigenous people with cancer (all cancers) that have investigated this issue. However, some studies have been conducted in specific cancer types. In comparing these studies, participants in our study utilised more community support services than that reported by gynaecological cancer survivors in Queensland (54%) [20]. The individual use of services in this study compared to the gynaecological cancer survivors study participants reported a higher use of information booklets (37%), similar internet use (23%) and lower use of telephone helplines (20%) [20]. Additionally, similar to the present study, Chisholm et al. [21] found that breast cancer patients treated in public hospitals utilized 50% or more of physiotherapist and social worker services. The higher use of community and allied health services reported by our participants may be explained by the strong association of service use and referral from a health care provider [20].

In general, no significant differences on service use were found by cancer type, age group, socio-economic status and place of residence. Females were more frequent users of ‘Information Sources’, and patients with higher levels of education were more frequent users of ‘Information resources’ and ‘Support Services’, whilst those with lower educational levels used more ‘Community Services’ more often.

Whilst not statistically significant a greater proportion of patients with breast and blood related cancers reported use of a wider range of health services than other cancer groups. Regarding breast cancer patients, the use of services might reflect a much greater volume of information available on breast cancer and a heightened awareness about the disease in the community. Additionally, there is perception that screening and treatment procedures for breast cancer are less invasive than those for other cancers e.g. cervical cancer. Research has shown that women especially in remote areas feel constrained to seek a Pap smear procedure. The reasons for this are many and include such issues as the patient may know the personnel who carry out the procedure (lack of privacy), [22] or the procedure is not clearly understood, or is carried out by a male professional. Patients with blood related cancer usually have rigorous monitoring of cell counts, blood transfusions, bone marrow transplantation, and control of infections that implies on constant medical appointments and hospital visits. These factors are likely to contribute to a higher proportion of these patients accessing different health services.

The demographic characteristics of the study population was similar to a previous study on cancer amongst Indigenous Australians [23] where Indigenous cancer cases were more likely to be women and under 59 years of age. However, the pattern of cancer types among the study sample differed from the patterns of cancers among Indigenous Queenslanders. In this study, breast and blood related were the most frequent cancer types, while the most commonly occurring cancers reported for Indigenous Queenslanders are lung and prostate among men, and breast and lung among women [2]. Nearly half of the patients included here were younger than 40 years of age at diagnosis; this accords with other studies reporting that cancer affects Indigenous Australian at younger ages than their non-Australian counterparts [5,23].

The Indigenous Health Liaison Officers (IHLOs) were reported as the most frequent service used. This is probably an indication of the availability of such services in the community and/or hospitals where these patients received treatment. Also, our finding reinforce the results of previous studies [16,24] that demonstrated Indigenous cancer patients have specific perceptions about quality of care and the cultural appropriateness of health services. Indigenous cancer patients have strong family and kin links that should be taken into account by care providers. The communication of health professionals and patients and decision making process appear to play a key role on the engagement of patients in the treatment [25]. There is increasing evidence indicating that the mainstream health delivery model is not the most culturally sensitive and comprehensive model for Indigenous patients in general (not specifically for cancer patients). The evidence suggests that collective community-governed health service delivery is a more appropriate model to overcome Indigenous health disadvantages [26]. Some evidence is also emerging overseas of community-based interventions to reduce cancer disparities among Alaska natives and American Indians [27].

It is important to highlight that less than one third of cancer patients in this study had completed high school or equivalent and that the participants level of Education was the most significant factor affecting health services utilization [16,24]. It is imperative that this be considered when planning cancer support services, and developing information sheets/brochures about cancer, cancer treatment and support for this group.

These principal findings should be considered in the context of the methodological features of the study. Strengths included the use of face-to-face interviews with trained Indigenous and non-Indigenous interviewers, use of a standardised data collection sheet, and the inclusion of multiple hospitals recruiting patients from a large geographic area (including patients who live in remote and very remote areas). A response rate of 57.7% of Indigenous cancer patients is a limitation of the study. Selection bias in our study may have resulted in participants being of higher socio-economic status than the general Indigenous population, and therefore more likely to access services compared to those not included in the study. For example, in the study sample about 30% of the participants had completed high school while in the 2006 census 23% of Australian Indigenous population had completed high school or equivalent [28]. Given the information about health service use was collected retrospectively, recall bias may potentially have affected the results. Nevertheless, there is no reason to believe that there was differential among the different groups e.g. types of cancer, age groups. In Australia, ethnicity is defined by self-assessment [29] and therefore not all Indigenous people with cancer may have been identified. There is some evidence that, occasionally, an Indigenous patient might be reluctant to identify themselves as such, or that hospital staff might not ask or assume their Indigenous status [30]. However, we believe steps taken in study design (e.g. face-to-face contact to confirm Indigenous status) minimized misclassification.

In Queensland, more than half of the Indigenous population live in outer regional, remote or very remote areas [13]. When diagnosed with cancer, they have to travel to a large city (e.g. Brisbane, Townsville) for their treatment. Indeed, over half the patients indicated they were away from their usual place of residence at the time of the study. In this regard, there are a few limitations in this study. Firstly, there is no differentiation between the use of services ‘at home’ or ‘whilst away from home for treatment or follow up care for their cancer’ and secondly, the questionnaire used a preset list of health services/workers. It is possible that our reported high use of services was mostly due to services accessed ‘whilst at a tertiary centre’ and not services available at their place of residence. Moreover, health services/workers not listed (e.g. the general practitioner) may be were less likely to be recalled.

However, often access is inhibited not only due to geographical distance but also cultural factors, and thus this can be experienced in an urban, rural or remote setting [31].

We have no information about health service use among cancer patients who did not receive any treatment. We know from previous reports, that Indigenous patients have lower treatment participant rates than their non-Indigenous counterparts. In a Queensland study, Indigenous cancer patients were 24% less likely to have surgery, 20% less likely to have chemotherapy and 9% less likely to receive radiotherapy [32]. In addition, for a large number of patients included in the study, the Indigenous Liaison Officer was the person referring patients to the study, so most patients included would have had access to an Indigenous Health Worker/Service. Lastly, given the challenges in recruiting Indigenous people with cancer in Queensland, even though in this context it may be considered a ‘large study’, nevertheless it has limited power to detect small differences between the groups with certainty; consequently, there may have been differences that the study did not detect. This is particularly relevant when we consider that remoteness did not affect utilisation in this study, it’s likely that in a study with more power we would detect these differences.

## Conclusion

Our findings suggest that factors such as accessibility (place of residence) and socioeconomic status were not significantly associated with health services utilization. The underlying factors affecting the health services utilization by Indigenous cancer patients remain not fully understood. There is a need for further systematic investigation into the health service utilization by Indigenous cancer patients. In particular, if patients accessed these services whilst tertiary care or back in their community; why mortality rates are much higher amongst Indigenous patients compared to non-Indigenous cancer patients despite the higher rate of access to services; and whilst Indigenous cancer patients reported a high use of health services their level of satisfaction with these services requires further clarification.

The study confirmed the importance of the Indigenous Health Liaison Officers’ and their role in supporting Indigenous cancer patients. However, it also highlights the need of ongoing education and information about the range of support services available to cancer patients generally so they can refer the patients to these services. Also initiatives of a more culturally friendly/appropriate model of care such as the patient navigator should be explored.

## Competing interest

The authors declare that they have no competing interests.

## Authors’ contributions

GG and PCV were involved in the study from the design phase to the final manuscript. CMB collected/analysed data and drafted the first manuscript. LW analysed data and reviewed the paper. All authors read and approved the final manuscript.

## Supplementary Material

Additional file 1 **Section 3:** Your use of community services. Click here for file
