# Supplementary material for: Health service utilization by indigenous cancer patients in Queensland: a descriptive study
Source: Int J Equity Health. 2012 Oct 10;11:57. doi: 10.1186/1475-9276-11-57 (PMC3522530; doi:10.1186/1475-9276-11-57)
Supplement: Additional file 1 — Section 3: Your use of community services. [file 1475-9276-11-57-S1.docx]

Table 1. Reported Health Services utilization by Indigenous cancer patients in Queensland.

| **Health Services Groups*** | Number | (%) |
| --- | --- | --- |
|  |  |  |
| **Group 1 - Indigenous Health Workers/Services** | **120** | **(76.4**) |
| Aboriginal Health Service | 55 | **(35.0**) |
| Indigenous Health Liaison Officer | 108 | (68.8) |
| Traditional Indigenous Practitioner | 3 | (1.9) |
|  |  |  |
| **Group 2 - Information Sources** | **111** | (**70.7**) |
| Information sheets/ Brochures | 105 | (66.9) |
| Internet information | 34 | (21.7) |
| Education Program/workshop | 29 | (18.5) |
|  |  |  |
| **Group 3 - Support Services** | **46** | (**29.3**) |
| Cancer helpline | 19 | (12.1) |
| Chaplain | 17 | (10.8) |
| Community-based support group | 8 | (5.1) |
| Internet-based support group | 5 | (3.2) |
| Peer support | 7 | (4.5) |
| Tele-based cancer counselling | 3 | (1.9) |
|  |  |  |
| **Group 4 - Community Services** | **70** | (**44.6**) |
| Home and Community Care Services (HACC) | 31 | (19.7) |
| Respite Care | 4 | (2.5) |
| Transport | 53 | (33.8) |
|  |  |  |
| **Group 5 - Allied Health Workers/Services** | **114** | (**72.6**) |
| Complementary medicine practitioner | 5 | (3.2) |
| Community Health Nurse | 34 | (21.7) |
| Dietician | 66 | (42.0) |
| Exercise physiologist | 10 | (6.4) |
| Mental health team | 03 | (1.9) |
| Pain specialist | 18 | (11.5) |
| Physiotherapist | 36 | (22.9) |
| Psychologist | 15 | (9.6) |
| Psychiatrist | 3 | (1.9) |
| Relaxation/meditation class | 6 | (3.8) |
| Social worker | 60 | (38.2) |
|  |  |  |
| **Group 6 - Others** | **24** | (**15.3**) |
| Cancer Care Coordinators/Cancer Council/Cancer Care Queensland | 20 | (12.7) |
| General practitioner | 3 | (1.9) |
| Occupational Therapist | 2 | (1.3) |
| Speech Therapist | 2 | (1.3) |
| Red Cross | 1 | (0.6) |
| Homeless Organization | 1 | (0.6) |
| Elders | 1 | (0.6) |
| Breast Care Association | 1 | (0.6) |
| **Total** | **157** | (**100.0)** |
|  |  |  |

*The patients could indicate the use of more than one health service.

**Percentage calculated over total number of patients (157).
